# Deviation from the recommended schedule: optimal dosing interval for a two-dose vaccination programme

**DOI:** 10.1098/rsos.231971

**Published:** 2024-07-03

**Authors:** Zhen Wang, Gergely Röst, Seyed M. Moghadas

**Affiliations:** ^1^ Agent-Based Modelling Laboratory, York University, Toronto, Ontario M3J 1P3, Canada; ^2^ National Laboratory for Health Security, University of Szeged, Szeged, Hungary

**Keywords:** epidemic dynamics, vaccination, integro-differential equations, simulation

## Abstract

Optimizing vaccination impact during an emerging disease becomes crucial when vaccine supply is limited, and robust protection requires multiple doses. Facing this challenge during the early stages of the COVID-19 vaccine deployment, a pivotal policy question arose: whether to administer a single dose to a larger proportion of the population by deferring the second dose, or to prioritize stronger protection for a smaller subset of the population with the established dosing interval from clinical trials. Using a delay-differential model and considering waning immunity and distribution capacity, we compared these strategies. We found that the efficacy of the first dose significantly influences the impact of delaying the second dose. Even for a relatively low efficacy of the first dose, a delayed strategy may outperform vaccination with the recommended dosing interval in reducing short-term hospitalizations and deaths despite increase in infections. The optimal delay, however, depends on the specific outcome measured and timelines within which the vaccination strategy is evaluated. We found transition lines for the relative reduction of infection, hospitalization and death below which vaccination with the recommended schedule is the preferred strategy. In a realistic parameter space, our results highlight scenarios in which the conclusions of previous studies are invalid.

## 1. Introduction

The challenges of vaccination programmes in the event of an emerging disease often extend beyond the availability of a safe and effective vaccine. By late 2020, the unprecedented pace of research and development had resulted in a number of highly efficacious vaccines against COVID-19, including AstraZeneca [1], Pfizer-BioNTech [2] and Moderna [3]. However, regional and global demand initially far surpassed the supply of these vaccines that required two doses to induce a high level of protection against COVID-19 and its severe outcomes [4]. Consequently, faced with vaccine shortages, a number of countries implemented vaccine strategies that prioritized high-risk individuals, but also deviated from the recommended between-dose interval based on the results of clinical trials [5–7]. These strategies aimed to lengthen the time interval between doses in order to vaccinate a larger proportion of the population with a single dose of available vaccines. In the absence of any evidence on the effectiveness of a vaccination programme with extended dosing interval, the underlying argument relied on the expectation that the impact of vaccination in mitigating disease burden would be enhanced when a larger proportion of the population is protected partially (with a single dose of vaccines) as opposed to fewer individuals having a high level of protection (with two doses of vaccines). For instance, a modelling study concluded that swiftly administering single-dose vaccination in England reduced the overall COVID-19 hospitalizations and deaths due to the rapid increase in partial immunity within the population [5].

While vaccination programmes progressed, numerous studies also investigated the effects of extended intervals between vaccine doses on immune responses, consistently finding that delaying the second dose improves immune protection compared to the recommended schedule [8,9]. However, these studies primarily focused on immune levels after the second dose, neglecting an examination of vaccine effectiveness during the time interval between the first and second doses. Despite a single dose offering moderate protection against disease outcomes [5,7,10], an extended delay in the second dose could lead to a significant number of infections, potentially offsetting the benefits of vaccinating more people with a single dose [7]. Previous modelling studies have shown that the impact of vaccination with a delayed second dose (DSD) would depend on the protection efficacy and the rate of waning immunity after the first dose [7,11]. Furthermore, the possibility of a variant emerging with increased transmissibility and/or immune evasion suggests that vaccination with prolonged between-dose intervals may be less effective than the recommended schedule in reducing the overall disease burden [11], even if immune protection after a DSD is enhanced.

In this study, we sought to investigate scenarios in which delaying the second dose of vaccines would result in a lower disease burden, measured in terms of the number of primary infections, hospitalizations and deaths during the time interval between the first and second doses. We developed a system of integro-differential equations to describe the dynamics of disease spread with a two-dose vaccination strategy. Our model incorporated variables such as waning vaccine-induced and naturally acquired immunity, as well as the capacity for vaccine distribution. While we parameterized the model within the context of the COVID-19 pandemic, the general framework can be applied to evaluate vaccination strategies for other diseases during an outbreak. Varying the parameters pertaining to the infection dynamics, we determined the optimal dosing interval as a function of vaccine efficacy against infection and outcomes by comparing the disease burden in delayed vaccination scenarios to that of administering two doses following the recommended schedule. Our results show that the optimal dosing interval depends not only on the transmissibility of the disease, but also on the vaccine efficacy parameters and the specific disease burden metric (i.e. infection, hospitalization or death) targeted for mitigation.

## 2. The model

**Figure 1.**
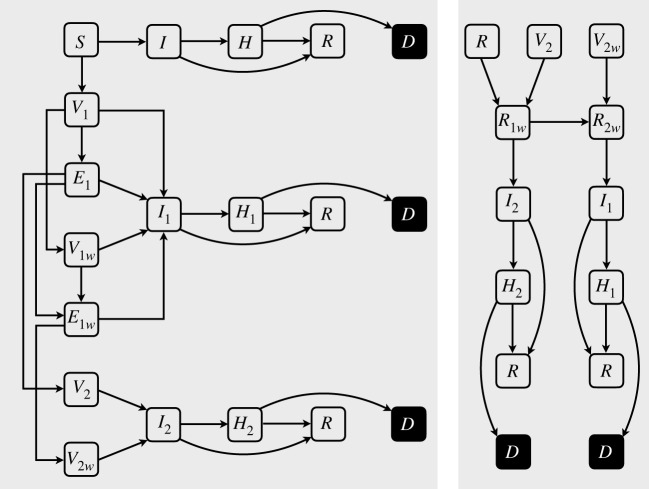
Schematic of disease dynamics in primary and secondary infections, with vaccination and waning immunity.

We developed the model framework based on the natural history of COVID-19 by dividing the population into several compartments (table 1) to represent the epidemiological statuses of unvaccinated individuals as susceptible (*S*), infected (*I*), hospitalized (*H*), recovered (*R*) and dead (*D*). Although the infectious stage of COVID-19 can be further divided into different classes (i.e. asymptomatic, presymptomatic and symptomatic), we combined these classes into a single compartment, noting that their infectiousness can be accounted for by adjusting the transmissibility of the disease in the model (figure 1). We assumed that infection transmission is possible only through contacts with infectious individuals who are not hospitalized, and that hospitalized individuals are completely isolated.

**Table 1.** Description of state variables in the model.

symbol	description
*S*(*t*)	susceptible population
*v*_1_(*t*, *a*)	population density of those who have received their first dose of vaccines *a* time-units ago
*v*_1*w*_(*t*, *a*)	population density of those who have received their first dose of vaccines *a* time-units ago but their immunity against infection has waned
*V*_1_(*t*)	individuals vaccinated with the first dose of vaccines
*V*_1*w*_(*t*)	individuals vaccinated with the first dose of vaccines in whom vaccine-induced immunity has waned
*E*_1_(*t*)	individuals eligible to receive the second dose of vaccines
*E*_1*w*_(*t*)	individuals eligible to receive the second dose of vaccines in whom immunity of the first dose has waned
*V*_2_(*t*)	individuals vaccinated with the second dose of vaccines
*V*_2*w*_(*t*)	individuals vaccinated with the second dose of vaccines in whom immunity of the second dose has waned
*I*(*t*)	infected individuals with no prior vaccination
*I*_1_(*t*)	infected individuals after vaccination with the first dose
*I*_2_(*t*)	infected individuals after vaccination with the second dose
*H*(*t*)	hospitalized individuals with no prior vaccination
*H*_1_(*t*)	hospitalized individuals after vaccination with the first dose
*H*_2_(*t*)	hospitalized individuals after vaccination with the second dose
*R*(*t*)	individuals recovered from primary infection
*R*_1*w*_(*t*)	recovered individuals in whom immunity against infection has partially waned
*R*_2*w*_(*t*)	recovered individuals in whom immunity against infection has completely waned
*D*(*t*)	dead individuals

With the introduction of vaccination, we added several compartments to represent the epidemiological statues of individuals as vaccinated with the first dose (*V*_1_) or the second dose (*V*_2_), infected after the first dose (*I*_1_) or the second dose (*I*_2_), with the corresponding hospitalization compartments of *H*_1_ and *H*_2_ (figure 1). We assumed that the risk of infection and disease outcomes are reduced by the efficacy of vaccines. Individuals who receive the first dose of vaccines acquire partial protection against infection and hospitalization, with reduced risk of death if they are hospitalized. The protection levels against infection and disease outcomes increase after receiving the second dose.

For recovered individuals, we considered a transient state of full protection against reinfection. To account for waning naturally acquired immunity, the model included two additional compartments of *R*_1*w*_ with partial protection and *R*_2*w*_ with no protection against reinfection (figure 1). We assumed that the protection level of individuals in *R*_1*w*_ against hospitalization and death is similar to those who have received two doses of vaccines. Since infection-acquired immunity reduces the risk of hospitalization and death for several months [12], individuals in *R*_2*w*_ were assigned a partial level of immunity against disease outcomes similar to those vaccinated with a single dose of vaccines, despite having no protection against infection. We also accounted for the waning vaccine-induced immunity after the first dose (*V*_1*w*_) and after the second dose (*V*_2*w*_), assuming a transient state of partial protection against infection and a longer-lasting protection against disease outcomes. Since we are investigating the epidemic dynamics over a relatively short timeframe compared to the average lifespan, the demographics of births and deaths were omitted.

To formulate the temporal evolution of the model compartments (table 1), we first derive expressions for the rate at which individuals in *V*_1_(*t*) and *V*_1*w*_(*t*) become eligible to receive the second dose. Let *a* be the time elapsed since vaccination with the first dose. We define *v*_1_(*t*, *a*) to represent the population density, with respect to *a* at time *t*, of individuals who received their first dose of vaccine *a* time units ago and are still (partially) protected against infection. We also define *v*_1*w*_(*t*, *a*) to represent the population density, with respect to *a* at time *t*, of individuals who received their first dose of vaccine *a* time units ago but their immunity against infection has already waned. Individuals in the *v*_1_(*t*, *a*) state transition to *v*_1*w*_(*t*, *a*) at an average rate (*ξ*_1_) of waning immunity acquired from vaccination. Considering these population densities for *a* ≤ *τ*, where *τ* is the actual dosing interval, the dynamics of infection and immunity can be expressed by

2.1 $$\begin{array}{cc} & \left(\frac{\partial }{\partial t}+\frac{\partial }{\partial a}\right)v_{1}(t,a)=-\beta (1-\epsilon_{1})\Lambda v_{1}(t,a)-\xi_{1}v_{1}(t,a)\\ \; & \left(\frac{\partial n}{\partial t}+\frac{\partial }{\partial a}\right)v_{1w}(t,a)=\xi_{1}v_{1}(t,a)-\beta \Lambda v_{1w}(t,a) \end{array}\}$$

where *Λ* = *I* + *I*_1_ + *I*_2_; *β* is the baseline transmission rate; $\epsilon_{1}$ is the vaccine efficacy against infection after the first dose; and *ξ*_1_ is the rate of waning vaccine-induced immunity against infection after the first dose. The boundary conditions are *v*_1_(*t*, 0) = *ρ*_1_(*t*), where *ρ*_1_(*t*) is the daily rate of vaccinating susceptible individuals with the first dose of vaccines, and *v*_1*w*_(*t*, 0) = 0. Since vaccination starts at time *t* = 0, we set *v*_1_(*t*, *a*) = *v*_1*w*_(*t*, *a*) = 0 for *a* ∈ [0, *τ*] at *t* = 0. Solving the first equation in (2.1) by integrating along characteristics gives

$$v_{1}(t,a)=v_{1}(t-a,0)e^{-\beta (1-\epsilon_{1})\int_{t-a}^{t}\Lambda (u)\;du-\xi_{1}a}.$$

In particular, those who have received their first dose of vaccine *τ* time-units ago (where *τ* is greater than or equal to the recommended dosing interval) move to a new compartment, denoted by *E*_1_, and may receive the second dose. Using the solution *v*_1_(*t*, *a*), and solving the second equation in (2.1) gives

$$v_{1w}(t,a)=\int_{0}^{a}\xi_{1}v_{1}(t-u,a-u)e^{-\beta \int_{t-u}^{t}\Lambda (s)ds}du.$$

Similarly, substituting *a* = *τ* in the solution *v*_1*w*_(*t*, *a*), we obtain

$$v_{1w}(t,\tau )=\int_{0}^{\tau }\xi_{1}\rho_{1}(t-\tau )e^{-\beta (1-\epsilon_{1})\int_{t-\tau }^{t-u}\Lambda (s)\;ds-\xi_{1}(\tau -u)}\;e^{-\beta \int_{t-u}^{t}\Lambda (s)\;ds}\;du,$$

which is the rate at which individuals in *V*_1*w*_ move to a new compartment, denoted by *E*_1*w*_, and may receive the second dose of vaccines. Any *τ* greater than the recommended dosing interval corresponds to a delay in administering the second dose. Summarizing the above, the model can be expressed by the following system of integro-differential equations:

$$\begin{array}{rl} & \frac{\text{d}S}{\text{d}t}=-\beta S\Lambda -\rho_{1}(t),\\ & \frac{\text{d}V_{1}}{\text{d}t}=\rho_{1}(t)-v_{1}(t,\tau )-\beta (1-\epsilon_{1})V_{1}\Lambda -\xi_{1}V_{1},\\ & \frac{\text{d}V_{1w}}{\text{d}t}=\xi_{1}V_{1}-v_{1w}(t,\tau )-\beta V_{1w}\Lambda ,\\ & \frac{\text{d}E_{1}}{\text{d}t}=v_{1}(t,\tau )-\beta (1-\epsilon_{1})E_{1}\Lambda -\xi_{1}E_{1}-\rho_{2}(t),\\ & \frac{\text{d}E_{1w}}{\text{d}t}=v_{1w}(t,\tau )+\xi_{1}E_{1}-\beta E_{1w}\Lambda -\rho_{2w}(t),\\ & \frac{\text{d}V_{2}}{\text{d}t}=\rho_{2}(t)-\beta (1-\epsilon_{2})V_{2}\Lambda -\xi_{2}V_{2},\\ & \frac{\text{d}V_{2w}}{\text{d}t}=\rho_{2w}(t)-\beta (1-\epsilon_{1})V_{2w}\Lambda -\xi_{1}V_{2w},\\ & \frac{\text{d}I}{\text{d}t}=\beta S\Lambda -[(1-\sigma )\gamma +\sigma h]I,\\ & \frac{\text{d}I_{1}}{\text{d}t}=\beta [(1-\epsilon_{1})(V_{1}+E_{1})+(V_{1w}+E_{1w})+R_{2w}]\Lambda -[(1-\sigma_{1})\gamma +\sigma_{1}h]I_{1},\\ & \frac{\text{d}I_{2}}{\text{d}t}=\beta [(1-\epsilon_{2})V_{2}+(1-\epsilon_{1})(V_{2w}+R_{1w})]\Lambda -[(1-\sigma_{2})\gamma +\sigma_{2}h]I_{2},\\ & \frac{\text{d}H}{\text{d}t}=\sigma hI-\mu H,\\ & \frac{\text{d}H_{1}}{\text{d}t}=\sigma_{1}hI_{1}-\mu H_{1},\\ & \frac{\text{d}H_{2}}{\text{d}t}=\sigma_{2}hI_{2}-\mu H_{2},\\ & \frac{\text{d}R}{\text{d}t}=(1-\sigma )\gamma I+(1-\sigma_{1})\gamma I_{1}+(1-\sigma_{2})\gamma I_{2}+(1-d)\mu (H+H_{1}+H_{2})\\ & \;+\eta_{1}d\mu H_{1}+\eta_{2}d\mu H_{2}-w_{1}R,\\ & \frac{\text{d}R_{1w}}{\text{d}t}=\xi_{2}V_{2}+w_{1}R-\beta (1-\epsilon_{1})R_{1w}\Lambda -w_{2}R_{1w},\\ & \frac{\text{d}R_{2w}}{\text{d}t}=\xi_{1}V_{2w}+w_{2}R_{1w}-\beta R_{2w}\Lambda \\ \mathrm{and}\;\; & \frac{\text{d}D}{\text{d}t}=d\mu H+(1-\eta_{1})d\mu H_{1}+(1-\eta_{2})d\mu H_{2}, \end{array}$$

where 1/*γ* is the infectious period; $\epsilon_{2}$ is the vaccine efficacy against infection after the second dose; 1/*h* is the length infectious period prior to hospitalization; 1/*μ* is the length of hospital stay; *d* represents the risk of death for hospitalized individuals without vaccination; *η*_1_ and *η*_2_ are the reduction in the risk of death for hospitalized individuals after the first and second doses of vaccines, respectively; *ξ*_2_ is the rate of waning vaccine-induced immunity against infection after the second dose; *w*_1_ and *w*_2_ are the rates of waning naturally acquired immunity against infection; and *ρ*_2_(*t*) and *ρ*_2*w*_(*t*) are the daily rates of vaccinating individuals in *E*_1_ and *E*_1*w*_ with the second dose, respectively. Denoting the number of vaccine doses available per day by D, we define the rates of second dose vaccination proportionally by

2.2 ρ2(t)=E1(t)E1(t)+E1w(t)κD ρ2w(t)=E1w(t)E1(t)+E1w(t)κD}

where *κ* is the proportion of vaccine doses at time *t* allocated towards second dose vaccination. The parameter *κ* varies depending on the number of individuals in *E*_1_ and *E*_1*w*_ at time *t*. We now define $\rho_{1}(t)=(1-\kappa (t))D$ as the rate of vaccination with the first dose.

In our model, individuals who are infected without any prior vaccination (*I*) will exit the infection state at two different rates corresponding to recovery and hospitalization. In order to parameterize the model with the estimated probability of hospitalization without vaccination, we introduced an auxiliary parameter *σ* for the exit rates. In this formulation, the probability of hospitalization (*α*) is expressed by

$$\alpha =\frac{\sigma h}{(1-\sigma )\gamma +\sigma h},$$

from which *σ* can be back-calculated by

2.3 $$\sigma =\frac{\alpha \gamma }{\alpha \gamma +(1-\alpha )h}.$$

Similarly, we used auxiliary parameters *σ*_1_ and *σ*_2_ for the exit rates from infection states *I*_1_ and *I*_2_ after vaccination with the first and second doses. Denoting the probabilities of hospitalization after vaccination with the first and second doses by *p*_1_ and *p*_2_, respectively, the auxiliary parameters satisfy the following relationships:

$$p_{1}=\frac{\;\sigma_{1}h}{(1-\sigma_{1})\gamma +\sigma_{1}h}\;\mathrm{and}\;p_{2}=\frac{\;\sigma_{2}h}{(1-\sigma_{2})\gamma +\sigma_{2}h}.$$

Given *δ*_1_ and *δ*_2_ for the efficacies of the first and second doses of vaccines against hospitalization, respectively, the probabilities of hospitalization are calculated by *p*_1_ = (1 − *δ*_1_)*α* and *p*_2_ = (1 − *δ*_2_)*α*, from which the auxiliary parameters can be back-calculated by

2.4 $$\sigma_{1}=\frac{\;p_{1}\gamma }{p_{1}\gamma +(1-p_{1})h}\;\mathrm{and}\;\sigma_{2}=\frac{\;p_{2}\gamma }{p_{2}\gamma +(1-p_{2})h}.$$

**Remark 2.1.**

In our model, since vaccination starts at time *t* = 0, we set the initial conditions for compartments involving the delay term to zero, that is *V*_1_(*t*) = *V*_1*w*_(*t*) = *E*_1_(*t*) = *E*_1*w*_(*t*) = *V*_2_(*t*) = *V*_2*w*_(*t*) = 0 for −*τ* ≤ *t* ≤ 0. Other compartments had a non-negative initial condition for simulations.

## 3. Simulation scenarios and parameterization

We discretized the model using a non-standard finite-difference method (electronic supplementary material) to simulate and compare the impact of two vaccination scenarios with: (i) broader protection when more susceptible individuals are vaccinated with the first dose in a DSD strategy; and (ii) higher protection in a smaller subset of the population when the second dose is administered according to the recommended schedule. Considering the early stages of the COVID-19 pandemic with the original SARS-CoV-2 strain as a case study, we set the recommended schedule with a waiting period of 21 days between the first and second doses [2,7]. To evaluate the DSD scenario, we assumed the second dose could be delayed up to 12 weeks from day 21 after the first dose.

We assumed a maximum of 400 vaccine doses are available and administered per day in a population of *N* = 100 000 individuals [7], with a 5% pre-existing immunity in the baseline simulations, and 20% in the secondary analysis (electronic supplementary material). We set 0 ≤ *κ* ≤ 0.9, which allocates a minimum 10% of the available doses at time *t* towards vaccination of individuals with the first dose. To calculate the transmission rate *β* with units of 1/(time × people), we used two reproduction numbers of R=1.8 [13] and R=1.1 [14], implicitly accounting for the effect of other control measures. Using the expression R=βN/γ, with *γ* = 1/5.5, we obtained *β* = 3.273 × 10^−6^ and *β* = 2 × 10^−6^, for R=1.8 and R=1.1, respectively. Given *α* = 0.025, *δ*_1_ = 0.75 and *δ*_2_ = 0.95 (table 2), and fixing 1/*h* = 3, we used the relations (2.3) and (2.4) to calculate *σ* = 0.0138, *σ*_1_ = 0.00342 and *σ*_2_ = 0.000682. We varied $\epsilon_{1}$ from 0.3 to 0.6, and considered two scenarios of $\epsilon_{2}=0.75$ and $\epsilon_{2}=0.9$. We also fixed the rates of waning protection by vaccination and natural infection to *ξ*_1_ = 1/90, *ξ*_2_ = 1/120, *w*_1_ = 1/120 and *w*_2_ = 1/90. Other parameter values are provided in table 2.

**Table 2.** Description of the model parameters and their values used for simulations.

symbol	description	value/range	source
$\epsilon_{1}$	efficacy of the first dose against infection	30–60%	[7,10]
$\epsilon_{2}$	efficacy of the second dose against infection	60–90%	[7,10]
*α*	probability of being hospitalized without vaccination	0.025	[15–17]
*δ*_1_	efficacy of the first dose against hospitalization	60–90%	[7,18]
*δ*_2_	efficacy of the second dose against hospitalization	95%	[2,7,18]
*η*_1_	efficacy of the first dose against death	60–90%	[19,20]
*η*_2_	efficacy of the second dose against death	90–98%	[19,20]
1/*γ*	average infectious period (days)	5.5	[21]
1/*h*	duration of infectious period before hospitalization (days)	2–4	[22]
1/*μ*	average length of hospital stay (days)	10	[17,23]
*d*	proportion of hospitalized individuals who die	12.5%	[22,24]
1/*ξ*_1_	average duration of protection against infection after the first dose (days)	60–90	[25,26]
1/*ξ*_2_	average duration of protection against infection after the second dose (days)	90–120	[25,26]
1/*w*_1_	average duration of full protection against infection following recovery (days)	90–120	assumed
1/*w*_2_	average duration of partial protection against infection following recovery (days)	60–90	assumed
*σ*	auxiliary parameter	0.0138	calculated by equation (2.3)
*σ*_1_	auxiliary parameter	varies	calculated by equation (2.4)
*σ*_2_	auxiliary parameter	0.000682	calculated by equation (2.4)

In order to determine the effect of a DSD strategy during the delayed period, we calculated the relative reduction (RR) using the following expression:

3.1 $$\text{RR}=\frac{\text{CI in recommended schedule}-\text{CI in DSD}}{\text{CI in recommended schedule}},$$

where CI is the cumulative incidence of primary infection over the period in which the second dose of vaccines was delayed. Similar expressions were used to calculate the RR for cumulative hospitalizations and deaths following the primary infection in the two scenarios of vaccination. A positive RR indicates that the DSD strategy outperforms the vaccination programme with the recommended schedule, while negative RR means that the recommended schedule performs better than the DSD strategy. The critical transition lines are characterized by RR = 0.

## 4. Results

### 4.1. Low transmissibility: R=1.1

**Figure 2.**
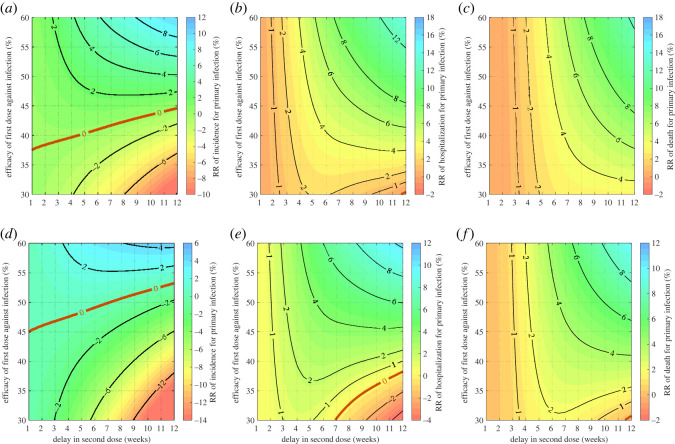
Relative reduction of total incidence (*a*,*d*), hospitalizations (*b*,*e*) and deaths (*c*,*f*) in primary infection during the delayed period for the second dose, with *β* = 2 × 10^−6^, *δ*_1_ = 0.75 and *κ* = 0.9. Vaccine efficacy against infection after the second dose was set to 75% (*a*–*c*) and 90% (*d*–*f*). Vaccination started at time *t* = 0 with *S*(0) = 94990, *I*(0) = 10, *R*(0) = 3000 and *R*_1*w*_(0) = 2000. The transition curve is represented by RR = 0 (red curve) below which the recommended strategy is preferred, and above which the DSD strategy is preferred.

We found that for a relatively low $\epsilon_{1}$, the recommended schedule is the preferred strategy for reducing the total incidence of primary infection (figure 2). For example, when $\epsilon_{1}=0.35$, delaying the second dose by 12-week results in over 6% increase in the incidence of primary infections with $\epsilon_{2}=0.75$ (figure 2*a*). With the same delay, the increase in primary infections under the DSD strategy compared to the recommended schedule exceeds 12% when $\epsilon_{2}=0.9$ (figure 2*d*). We observed a transition line RR = 0, above which a DSD strategy outperforms the recommended schedule in reducing the incidence of primary infection. For instance, when $\epsilon_{1}=0.55$, the cumulative incidence of primary infection in the DSD strategy with a delay of 12 weeks could be reduced by at least 7% with $\epsilon_{2}=0.75$, and 1% with $\epsilon_{2}=0.9$, compared to the recommended schedule (figure 2*a*,*d*).

For hospitalizations and deaths following the primary infection, a DSD strategy outperformed the recommended schedule in most of the parameter regions (figure 2). When $\epsilon_{2}=0.75$, delaying the second dose resulted in fewer hospitalizations and deaths during the delayed period, even for a relatively low $\epsilon_{1}$ despite an increase in the incidence of primary infection below the transition line RR = 0 (figure 2*b*,*c*). This result can be explained by a higher proportion of the population being vaccinated with the first dose as the delay in the second dose increases, providing a broader protection against disease outcomes including hospitalizations and deaths. When $\epsilon_{2}$ increased to 0.9, we observed a transition line RR = 0 for $\epsilon_{1}<0.38$, below which the total number of hospitalizations was higher (RR < 0) in the DSD strategy when the delay in the second dose exceeded seven weeks (figure 2*e*). However, the total number of deaths in the DSD strategy was still lower (RR > 0) or comparable to the recommended schedule (figure 2*f*).

#### 4.1.1. Strategy evaluation during the first 100 days

**Figure 3.**
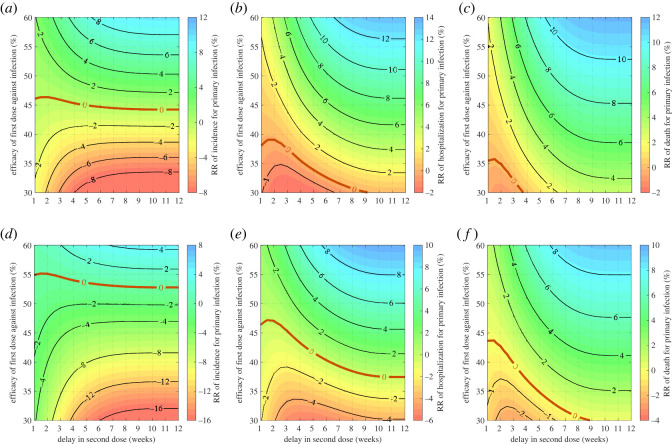
Relative reduction of total incidence (*a*,*d*), hospitalizations (*b*,*e*) and deaths (*c*,*f*) following primary infection over the first 100 days of the epidemic, with *β* = 2 × 10^−6^, *δ*_1_ = 0.75 and *κ* = 0.9. Vaccine efficacy against infection after the second dose was set to 75% (*a*–*c*) and 90% (*d*–*f*). Vaccination started at time *t* = 0 with *S*(0) = 94990, *I*(0) = 10, *R*(0) = 3000 and *R*_1*w*_(0) = 2000. The transition curve is represented by RR = 0 (red curve) below which the recommended strategy is preferred, and above which the DSD strategy is preferred.

We further evaluated the DSD strategy by computing the RR over the first 100 days of the epidemic. Similar to the scenarios during the delayed period, we found transition lines of RR = 0 below which the recommended schedule resulted in a lower cumulative incidence compared to the DSD strategy (figure 3*a*,*d*). We also observed transition lines RR = 0 when the total number of hospitalizations and deaths in the DSD strategy were compared to those in the recommended schedule. Above the transition lines, increasing delay in administering the second dose reduced overall hospitalizations and deaths during the first 100 days of the epidemic (figure 3*b*,*c*,*e*,*f*). However, the RR patterns below the transition lines are nonlinear, indicating a similar relative increase in the total number of hospitalizations and deaths for different delays with a relatively small $\epsilon_{1}$. For example, when $\epsilon_{1}=0.35$ and $\epsilon_{2}=0.9$, both two- and six-week delays in DSD strategy generated 2.7% higher number of hospitalizations over 100 days compared to the recommended schedule (figure 3*e*). This highlights the importance of the metric used for decisions to delay the second dose. In this scenario, a two-week delay increases the cumulative primary infections by 5.3%, which is lower than 12.2% increase with a six-week delay. However, a two-week delay increases the cumulative number of deaths by 1.4%, while a six-week delay reduces deaths by 0.7%, outperforming vaccination with the recommended schedule.

### 4.2. High transmissibility: R=1.8

**Figure 4.**
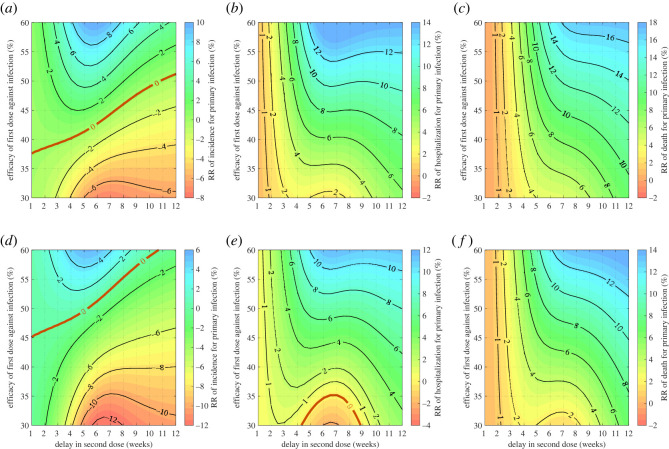
Relative reduction of total incidence (*a*,*d*), hospitalizations (*b*,*e*) and deaths (*c*,*f*) in primary infection during the delayed period for the second dose, with *β* = 3.273 × 10^−6^, *δ*_1_ = 0.75 and *κ* = 0.9. Vaccine efficacy against infection after the second dose was set to 75% (*a*–*c*) and 90% (*d*–*f*). Vaccination started at time *t* = 0 with *S*(0) = 94990, *I*(0) = 10, *R*(0) = 3000 and *R*_1*w*_(0) = 2000. The transition curve is represented by RR = 0 (red curve) below which the recommended strategy is preferred, and above which the DSD strategy is preferred.

Similar to the case of low transmissiblility with R=1.1, we found transition lines below which adhering to the recommended vaccination schedule yields fewer infections than the DSD strategy during the delayed period (figure 4). Above the transition line, the relationship between RR and vaccine efficacy exhibits nonlinearity, highlighting instances where the DSD strategy can lead to equivalent reductions in the incidence of infection for different delays in administering the second dose. For example, when $\epsilon_{1}=0.55$ and $\epsilon_{2}=0.75$, both a four- and seven-week delay in the DSD strategy resulted in approximately 6% lower number of infections over the delayed period compared to the recommended schedule. This result shows that, in certain conditions, partial protection in greater number of vaccinated individuals can be equivalent in its impact to the heightened protection of fewer vaccinated individuals. Notably, there is an optimal delay in the DSD strategy that leads to the maximum reduction in infections (figure 4*a*,*d*). For example, when $\epsilon_{1}=0.55$ and $\epsilon_{2}=0.75$, a 5.5-week delay renders the optimal DSD strategy, resulting in a 7.3% reduction in primary infections compared to the recommended schedule. When $\epsilon_{1}=0.55$ and $\epsilon_{2}=0.9$, the optimal delay in the DSD strategy was 4.5 weeks with a 2.9% reduction in primary infections. Below the transition lines (RR = 0) for incidence, the DSD strategy may still outperform the recommended schedule in reducing hospitalizations and deaths (figure 4*b*,*c*,*e*,*f*).

#### 4.2.1. Strategy evaluation during the first 100 days

**Figure 5.**
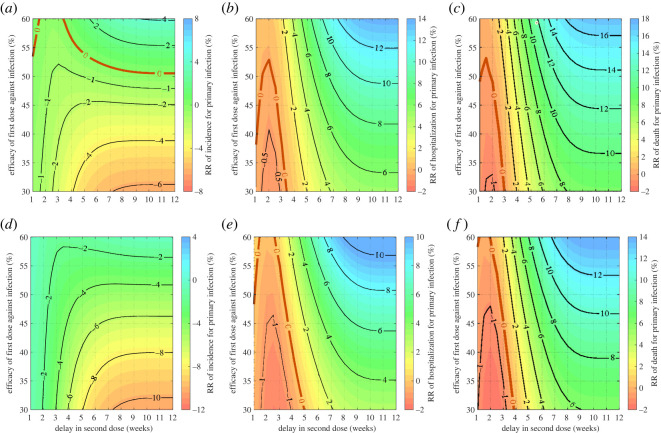
Relative reduction of total incidence (*a*,*d*), hospitalizations (*b*,*e*) and deaths (*c*,*f*) in primary infection over the first 100 days of the epidemic, with *β* = 3.273 × 10^−6^, *δ*_1_ = 0.75 and *κ* = 0.9. Vaccine efficacy against infection after the second dose was set to 75% (*a*–*c*) and 90% (*d*–*f*). Vaccination started at time *t* = 0 with *S*(0) = 94990, *I*(0) = 10, *R*(0) = 3000 and *R*_1*w*_(0) = 2000. The transition curve is represented by RR = 0 (red curve) below which the recommended strategy is preferred, and above which the DSD strategy is preferred.

Evaluating the DSD strategy over the first 100 days of the epidemic (figure 5), we identified transition lines of RR = 0 akin to those observed with R=1.1. However, compared to the scenario with lower transmissibility (i.e. R=1.1), the transition lines for incidence were shifted upward (figure 5*a*,*d*). Consequently, the parameter space in which the DSD strategy could outperform the recommended schedule is more constrained when considering the efficacy of the first dose, as opposed to the scenario with lower transmissibility. Notably, when $\epsilon_{2}=0.9$, any delay in the second dose resulted in an increase in infections.

The transition lines for hospitalizations and deaths have similarly shifted upward in the direction of increasing efficacy of the first dose, albeit within a more limited timeframe for delaying the second dose (figure 5*b*,*c*,*e*,*f*). For instance, when $\epsilon_{1}=0.35$ and $\epsilon_{2}=0.75$, any delay less than three weeks in the DSD strategy leads to an increase in hospitalizations compared to the recommended schedule.

### 4.3. Secondary analyses

Simulating the model with lower and upper bounds of the vaccine efficacy against hospitalization and death after the first dose, and with a higher level of pre-existing immunity (i.e. 20%) in the population at the start of vaccination (electronic supplementary material, figures S1–S12), we derived qualitatively similar patterns for the RR during both the delayed period and the first 100 days of the epidemic. Similar observations were made when simulating the model with the lower bounds of rates for waning protection (table 2), and using a reduced daily number of vaccine doses (specifically, 300 doses per day). These findings imply that the reduction in hospitalizations and deaths achieved with the DSD strategy during the delayed period may not necessarily translate to a lower overall outcome over an extended timeframe during the epidemic, especially when considering a relatively low vaccine efficacy of the first dose against infection.

## 5. Discussion

Vaccination has proven to be the most effective public health tool in curbing disease spread and mitigating its impact. To enhance the effectiveness of vaccination, especially in the face of limited vaccine availability, strategic utilization of existing doses is crucial. In this study, we employed a mathematical model to investigate the implications of delaying the second dose within a two-dose vaccination programme. Our simulations, based on parameters estimated for COVID-19, reveal that the efficacy of the first dose significantly influences the outcomes of delaying the second dose. We also demonstrated that the short-term impact—observable within the delayed period—on reducing the incidence, hospitalization, and death may be different from the longer-term effects of a DSD strategy.

A previous modelling study examining the delay of the second dose in COVID-19 vaccination concludes that a DSD strategy with the longest possible delay would be optimal if the efficacy of the second dose is less than or equal to twice the efficacy of the first dose [11]. However, our findings indicate that the validity of this conclusion may depend on the specific outcome being measured (incidence, hospitalization or death) and the timelines within which the vaccination strategy is evaluated. For example, as illustrated in our simulations (figures 2), the DSD strategy outperforms the recommended schedule in reducing hospitalizations and deaths during the delayed period even when $\epsilon_{2}>2\epsilon_{1}$. Notably, with relatively high transmissibility (i.e. R=1.8), the longest delay in the DSD strategy may result in higher infections even when $\epsilon_{2}<2\epsilon_{1}$. For instance, in the scenario where $0.45<\epsilon_{1}<0.5$, a short delay in administering the second dose proves to be more effective in reducing the total infections than the recommended schedule (figure 4*a*,*d*). However, as the delay in second dose administration increases, RR crosses the transition line, making vaccination with the recommended schedule more effective in infection reduction. This is notwithstanding the fact that the DSD strategy consistently outperforms the recommended schedule in reducing hospitalization and deaths for any delay (figure 4*b*,*c*,*e*,*f*).

Although our model was specifically designed to examine scenarios within the context of the COVID-19 pandemic, the general framework outlined here holds the promise for investigating other diseases for which a two-dose vaccine strategy is recommended (e.g. Mpox [27]). Adapting the model for such purposes may require modifications to the model structure and parameterization, based on the specific disease’s natural history and transmission dynamics. It is worth highlighting that the model is designed to investigate the scenarios involving vaccine distribution during an outbreak, potentially constrained by limited supply.

While our study offers a modelling framework to evaluate the effect of delaying the second dose, it is subject to certain limitations. First, we adopted a simplified, homogeneous setting, overlooking the complexity of transmission dynamics and outcomes that are often influenced by factors such as contact patterns, age and risk factors. Second, we simulated the model with only one vaccine type, without considering potential variations in efficacy at the individual level, or the simultaneous deployment of multiple vaccines with distinct efficacies and dosing intervals. Third, our analysis implicitly accounted for the effect of other control measures on the reproduction number. If the disease is highly transmissible, the speed of propagation will likely outpace the effect of vaccination as seen during the Omicron wave of the COVID-19 pandemic. Fourth, by gauging protection solely in terms of vaccine efficacy, we did not account for potential increase in protection against infection or disease outcomes post second dose [8,9]. However, the protection level of vaccine-induced immunity after a delayed second dose is unlikely to have a significant effect on our results for evaluating the DSD strategy within the delayed period. Finally, we did not consider the potential for increased protection and extended hybrid immunity as a result of infection post-vaccination. Under these limitations, we underscore the qualitative insights provided by our study, emphasizing the importance of timelines and specific outcomes in the evaluation of vaccination strategies.

## Data Availability

The parameters and computational code for reproducibility of the results are available at https://github.com/ABM-Lab/delay-vaccination. Supplementary material is available online [28].
